# Breathing-Zone Exposure to Aromatic Volatile Organic Compounds in Surgical Smoke During Transurethral Resection of Bladder Tumor: A Prospective Paired Monitoring Study

**DOI:** 10.3390/toxics14020130

**Published:** 2026-01-29

**Authors:** Seon Beom Jo, Sun Tae Ahn, Mi Mi Oh, Soo Ho Shim, Cheong Mo Ahn, Seul Gi Oh, Jong Wook Kim

**Affiliations:** 1 Department of Urology, Korea University Guro Hospital, Korea University College of Medicine, Seoul 08308, Republic of Korea; rhfughfkddl@naver.com (S.B.J.); asturology@gmail.com (S.T.A.); mamah77@paran.com (M.M.O.); 2 Blue squad Co., Ltd., Gangneung 25601, Republic of Korea; sooho.shim@bluesquad.co.kr (S.H.S.); bluesquad@bluesquad.co.kr (C.M.A.); summer@bluesquad.co.kr (S.G.O.)

**Keywords:** surgical smoke, TURBT, volatile organic compounds, BTEXS, formaldehyde, breathing-zone exposure, occupational exposure

## Abstract

(1) Background: Energy-based transurethral resection of bladder tumor (TURBT) generates surgical smoke that may contain hazardous volatile organic compounds (VOCs), yet surgeon breathing-zone exposure during transurethral surgery remains insufficiently characterized. (2) Methods: We conducted a prospective paired-exposure study during 28 TURBT procedures over 10 operating days using personal sampling at the surgeon’s breathing zone and simultaneous intraoperative background sampling at three predefined locations (~1.5 m from the surgeon). VOCs were measured by active sampling onto Tenax TA sorbent tubes followed by thermal desorption Gas Chromatography–Mass Spectrometry (GC–MS), and formaldehyde was measured by 2,4-dinitrophenylhydrazine (DNPH) cartridges with high-performance liquid chromatography/ultraviolet detection (HPLC/UV). Breathing-zone versus background contrasts were summarized as paired geometric mean ratios (GMRs), and a dose index was calculated as concentration × operative time (µg·h/m^3^). (3) Results: Breathing-zone concentrations consistently exceeded background levels, including total VOCs (GMR 4.31; 95% CI 2.92–6.38), ΣBTEXS (sum of benzene, toluene, ethylbenzene, xylenes, and styrene; GMR 2.10; 1.69–2.60), and styrene (GMR 8.51; 6.25–11.60); formaldehyde showed a smaller but significant elevation (GMR 1.20; 1.07–1.35). ΣBTEXS dose increased with operative time (Spearman ρ = 0.80, *p* < 0.001) and resection mass where available (ρ = 0.62, *p* = 0.0038; *n* = 20) and scaled with operative time (β = 0.86; R^2^ = 0.69; *n* = 28). (4) Conclusions: TURBT is associated with marked enrichment of aromatic VOCs in the surgeon’s breathing zone, supporting routine implementation of effective source-level smoke evacuation and filtration to reduce occupational exposure.

## 1. Introduction

Energy-based instruments used in transurethral endoscopic urologic surgery—such as transurethral resection of bladder tumor (TURBT) and transurethral resection of the prostate (TURP)—generate “surgical smoke,” a complex aerosol of gases and particles produced by thermal decomposition and vaporization of tissue. Chemical analyses consistently show volatile organic compounds (VOCs)—including benzene, toluene, ethylbenzene, and xylenes (BTEX)—as well as aldehydes, carbon monoxide (CO), and other polycyclic aromatic compounds, admixed with cellular and biologic debris. At the same time, aerosol-physics studies report particle sizes spanning the ultrafine to coarse range, with device- and tissue-dependent modes and concentrations [1,2,3,4]. Electrosurgery tends to produce a higher fraction of ultrafine particles, whereas ultrasonic and some laser devices generate larger modes; these differences have implications for respiratory deposition and endoscopic visibility [4,5]. In urology, multiple studies have identified hazardous gases liberated during TURP and TURBT. A preliminary endoscopic series demonstrated high short-range CO peaks and a complex hydrocarbon mixture during transurethral procedures [6]. Targeted gas chromatography/mass spectrometry (GC-MS) subsequently detected carcinogens during transurethral prostate resection or vaporization (e.g., acrylonitrile and 1,3-butadiene) and BTEX species during bladder tumor resections [7,8,9]. Taken together, these data suggest a characteristic aromatic VOC signature during TURBT. Given these hazards, contemporary guidance emphasizes local exhaust ventilation (LEV) at the source, effective filtration, and respiratory protection as a secondary control when capture is suboptimal [10,11,12,13,14]. The Association of periOperative Registered Nurses (AORN) 2022 Guideline in Practice operationalizes the hierarchy of controls for surgical smoke and recommends evacuating and filtering all smoke at the source, including during minimally invasive surgery (MIS), preferentially with ultra-low particulate air (ULPA) filtration plus activated carbon; personal protective equipment (e.g., a National Institute for Occupational Safety and Health (NIOSH)-approved filtering facepiece respirator (N95)) is a backstop, not a primary control [11]. Bench and clinical evaluations of built-in filtration/exsufflation ports during MIS also show reductions in several VOCs—with variable performance for formaldehyde—underscoring both progress and remaining engineering gaps [12].

Despite decades of work, key exposure questions remain under-characterized in transurethral surgery. Many urologic studies have sampled within irrigation circuits or near the scope tip rather than in the surgeon’s breathing zone, limiting inference about personal exposure [6,7,8,9]. Time–distance studies in urologic operating rooms (ORs) demonstrate sharp spikes in particulate matter ≤ 2.5 μm (PM2.5) at the operator position within seconds of activation and only partial reduction with wall suction, hinting that proximity and capture efficiency dominate dose [15]. Against this backdrop, we prospectively quantified breathing-zone exposure to total volatile organic compounds (TVOCs) and selected analytes (benzene, toluene, ethylbenzene, xylenes, styrene, and formaldehyde) during TURBT, compared these with paired perioperative background measurements, and modeled whether operative time and resection weight predict cumulative dose.

## 2. Materials and Methods

### 2.1. Study Design and Sampling Layout

**Figure 1:**
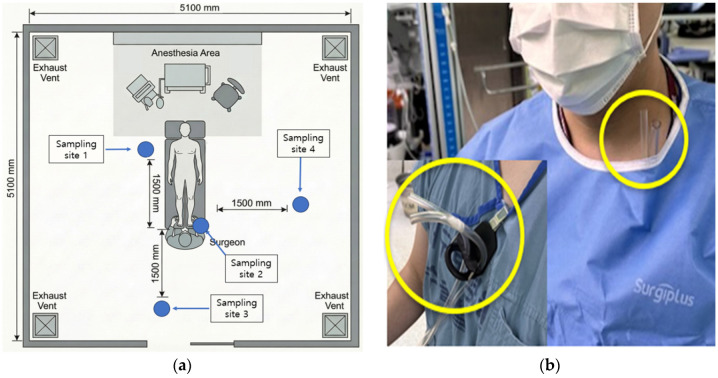
(**a**) Spatial configuration of air-sampling locations in the operating room. Sampling was performed at four sites: the surgeon’s breathing zone (site 2) and three background locations positioned 1.5 m from the surgeon (sites 1, 3, and 4). The three background sites were treated as spatial replicates, and the geometric mean of their concentrations was used as the background concentration for paired analyses. The schematic depicts operating room dimensions, patient position, anesthesia area, and exhaust vents. (**b**) Configuration of the personal air-sampling device at the surgeon’s breathing zone. The device was secured to the neck before gowning, with only the air-inlet tube exposed and positioned adjacent to the breathing zone to sample breathing-zone air.

We performed a prospective, paired-exposure study during transurethral resection of a bladder tumor. Personal samplers were clipped at the surgeon’s breathing zone (collar; ~20 cm from the nose–mouth), and background samplers were positioned at three predefined sites, approximately 1.5 m from the surgeon (~1.5 m above the floor), to represent the intraoperative room background (Figure 1a). All procedures were performed in a positive-pressure operating room with routine ventilation (approximately 20–25 air changes per hour, per facility standard), and no local smoke-evacuation device was used. The personal sampler configuration (device secured at the neck before gowning, with only the inlet tube exposed adjacent to the breathing zone after gowning) is shown in Figure 1b. During the intraoperative period, pumps ran continuously whenever surgery was in progress. Session blanks were collected. Pumps were pre-/post-calibrated (±5%) with a primary flow calibrator.

### 2.2. VOCs Thermal Desorption Gas Chromatography–Mass Spectrometry (GC–MS)

Sampling: Air was actively drawn through two Tenax TA sections (APK sorbent tubes; KNR Co., Ltd., Seongnam-si, Republic of Korea) at 100 mL·min^−1^, with continuous operation throughout the intraoperative window at each location; consequently, the total sampled volume varied by case length and was recorded. Tubes were capped and refrigerated (≤14 days). Backup sections were analyzed for breakthrough. Procedures followed the U.S. Environmental Protection Agency (EPA) Method TO-17 and ASTM International (ASTM; formerly the American Society for Testing and Materials) D6196, with reporting aligned to the International Organization for Standardization (ISO) standard ISO 16000-6 where applicable [16,17,18].

Analysis: Tubes were analyzed using a TD100-xr thermal desorber (Markes International, Llantrisant, UK) coupled to an Agilent 7890B/5977B GC–MS (Agilent Technologies, Santa Clara, CA, USA) equipped with an HP-1MS capillary column (60 m × 0.32 mm × 0.25 µm; Agilent Technologies, Santa Clara, CA, USA), operated in electron ionization (EI) full-scan mode under a single validated program compliant with TO-17/ASTM D6196 [16,17]. Data acquisition and processing were performed using Agilent workstation software (version B.08.00; Agilent Technologies, Santa Clara, CA, USA).

Targets and summary metrics: We quantified benzene, toluene, ethylbenzene, m- and p-xylene, o-xylene, styrene, and other routinely detected species (e.g., 2-butanone, 1-butanol, trimethylbenzenes, and C11–C14 alkanes).

For summary reporting, total volatile organic compounds (TVOC) refers to the ISO-defined TVOC (ISO-TVOC; C6–C16 and toluene equivalents) where full-scan integration is permitted [18]. In addition, we report an aromatic VOC indicator (ΣBTEXS), defined as the toluene-equivalent sum of benzene, toluene, ethylbenzene, and xylenes (BTEX) plus styrene. Calibration used external multi-point tube standards (≈50–500 ng) prepared from custom VOC standards (S-19044-R1 and S-112247; AccuStandard, Inc., New Haven, CT, USA) in methanol (Fisherbrand, cat. HCB121; Thermo Fisher Scientific, Waltham, MA, USA). Field blanks and lab duplicates were ≥10% per batch; method detection limits (MDLs) were established from seven near-limit-of-detection (LOD) replicates (pre-specified precision/accuracy/recovery criteria).

### 2.3. Formaldehyde (2,4-Dinitrophenylhydrazine (DNPH)–High-Performance Liquid Chromatography (HPLC) with Ultraviolet (UV) Detection; DNPH–HPLC/UV)

Sampling: 2,4-dinitrophenylhydrazine (DNPH)-coated silica cartridges (815K; EvergreenTop Co., Ltd., Pocheon-si, Republic of Korea) were operated at 500 mL·min^−1^ continuously across the same intraoperative window, yielding case-length–dependent air volumes, with an in-line potassium iodide (KI) ozone scrubber (E-OZ5; EvergreenTop Co., Ltd., Pocheon-si, Republic of Korea), per the U.S. Environmental Protection Agency (EPA) Method TO-11A [19].

Analysis: Extracts were analyzed using an Agilent 1260 Infinity II high-performance liquid chromatography (HPLC) system (Agilent Technologies, Santa Clara, CA, USA) with an EC-C18 column (4 µm, 4.6 × 150 mm; Agilent Technologies, Santa Clara, CA, USA), isocratic acetonitrile/water (ACN/H_2_O) (acetonitrile, P1D203; water, NAH105; J.T. Baker (Avantor, Radnor, PA, USA)), and ultraviolet (UV) detection at 360 nm. Formaldehyde–DNPH was quantified using a certified reference material (CRM47177; Formaldehyde-2,4-DNPH in acetonitrile; Supelco, Bellefonte, PA, USA) and converted to µg/m^3^ at 25 °C, 1 atmosphere (atm); the procedure aligns with the International Organization for Standardization (ISO) standard ISO 16000-3 and the National Institute for Occupational Safety and Health (NIOSH) Manual of Analytical Methods (NMAM), Method 2016 [20,21]. Data acquisition and processing were performed using Agilent chromatography data system software (version D.07.40; Agilent Technologies, Santa Clara, CA, USA).

### 2.4. Data Handling and Statistics

Analytes were blank-corrected and expressed as µg/m^3^ (temperature- and pressure-corrected volumes). Non-detects were imputed as the limit of quantitation (LOQ)/√2 for summaries. Breathing-zone versus background contrasts are presented as geometric mean ratios (GMRs) with 95% confidence intervals based on log-transformed paired ratios, where the background concentration for each procedure was defined as the geometric mean across the three background sites (sites 1, 3, and 4); two-sided *p*-values were obtained from paired *t*-tests on ln ratios. Operative time for exposure calculations was defined as pump run time during the intraoperative window (derived from sampled air volume at a fixed flow rate). A time-integrated exposure index (“dose index”) was calculated for each analyte as concentration × operative time (µg·h/m^3^); dividing by operative time yields the mean concentration, which is already reported separately. All statistical analyses and data visualization were performed in Python (version 3.10.13; Python Software Foundation, Wilmington, DE, USA) using NumPy (version 1.26.4), pandas (version 2.2.0), SciPy (version 1.12.0), statsmodels (version 0.14.1), and Matplotlib (version 3.10.8).

## 3. Results

### 3.1. Case Characteristics

A total of 28 TURBT procedures were sampled across 10 operating days (Table 1). Median operative time was 51 min (interquartile range (IQR) 39–73; range 15–138; mean 59.2 ± 31.0). Resection mass was available for 20/28 cases, with a median of 7.0 g (IQR 5.0–15.2; range 2.0–23.0; mean 9.85 ± 6.43), leaving 8 cases with missing resection mass (Table 1).

### 3.2. Breathing-Zone Versus Background Concentrations

**Figure 2:**
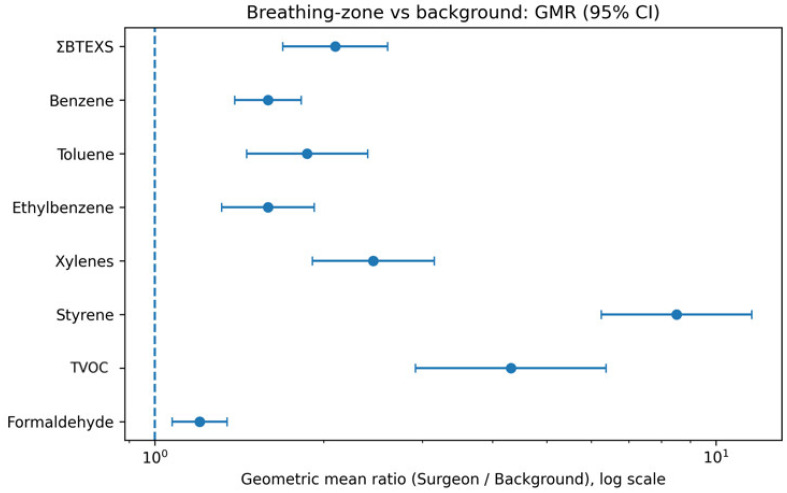
Forest plot comparing surgeon breathing-zone to intraoperative background concentrations during transurethral resection of bladder tumor (TURBT). Points show geometric mean ratios (GMRs) with 95% confidence intervals (CIs); the dashed line at GMR = 1 indicates no difference, and values > 1 indicate higher breathing-zone concentrations. Analytes include TVOC, ΣBTEXS, individual BTEX compounds, styrene, and formaldehyde. Abbreviations: TVOC, total volatile organic compounds; ΣBTEXS, sum of benzene, toluene, ethylbenzene, xylenes, and styrene (toluene-equivalents); BTEX, benzene, toluene, ethylbenzene, and xylenes.

Across target analytes, surgeon breathing-zone concentrations were higher than intraoperative background levels (Table 2; Figure 2). TVOC was elevated 4.31-fold in the breathing zone compared with background (GMR 4.31; 95% CI 2.92–6.38; *p* < 0.0001). The primary aromatic metric ΣBTEXS was 2.10-fold higher (1.69–2.60; *p* < 0.0001), with the largest contrast observed for styrene (GMR 8.51; 6.25–11.60; *p* < 0.0001). Individual BTEX species were also consistently elevated (e.g., benzene GMR 1.59; toluene 1.87; ethylbenzene 1.59; total xylenes 2.45; all *p* < 0.0001). Formaldehyde showed a statistically significant but smaller elevation (GMR 1.20; 1.07–1.35; *p* = 0.0023) (Table 2).

### 3.3. Consistency Across Procedures and Aromatic Mixture Profile

At the case level, the breathing-zone concentration exceeded background concentration in the majority of procedures: 27/28 (96.4%) for TVOC, 26/28 (92.9%) for ΣBTEXS, 27/28 (96.4%) for styrene, and 24/28 (85.7%) for formaldehyde. Median paired ratios (breathing zone/background) were 4.21 [IQR 1.93–7.30] for TVOC, 1.99 [1.39–2.92] for ΣBTEXS, 8.79 [5.24–14.72] for styrene, and 1.13 [1.07–1.20] for formaldehyde, indicating that the observed GMRs reflect a consistent proximity effect rather than a small number of outliers.

Within ΣBTEXS, toluene and xylenes accounted for the largest fractions in both locations; however, styrene contributed a larger fraction of ΣBTEXS in the breathing zone than in the background (median 6.4% vs. 1.4%), consistent with near-source enrichment of styrene. Formaldehyde concentrations were strongly correlated between breathing-zone and background samples (Spearman’s ρ = 0.89, *p* < 0.001), suggesting a relatively stronger room-level component than that of the more proximity-dependent aromatic VOCs.

Exploratory full-scan TD GC-MS species also suggested enrichment of several hydrocarbons and substituted aromatics in the breathing zone, including undecane (GMR 5.44; 95% CI 3.10–9.55) and 1,2,3-trimethylbenzene (GMR 3.46; 2.18–5.49), whereas some oxygenated VOCs (e.g., 2-butanone and 1-butanol) did not show consistent elevation.

### 3.4. Dose Index and Associations with Operative Time and Resection Mass

Dose indices (concentration × operative time; µg·h/m^3^) increased with operative time across all analytes (Table 3). Associations with operative time were strongest for ΣBTEXS dose (Spearman ρ = 0.80, *p* < 0.001), ethylbenzene dose (ρ = 0.81, *p* < 0.001), styrene dose (ρ = 0.79, *p* < 0.001), formaldehyde dose (ρ = 0.79, *p* < 0.001), and xylenes dose (ρ = 0.71, *p* < 0.001) (Table 3).

Resection mass (available *n* = 20) showed moderate associations with cumulative dose for several analytes, including the ΣBTEXS dose (ρ = 0.62, *p* = 0.0038), ethylbenzene dose (ρ = 0.59, *p* = 0.0063), styrene dose (ρ = 0.59, *p* = 0.0061), xylenes dose (ρ = 0.56, *p* = 0.0098), and formaldehyde dose (ρ = 0.48, *p* = 0.032), whereas correlations for TVOC, benzene, and toluene dose did not reach statistical significance in this dataset (Table 3).

**Figure 3:**
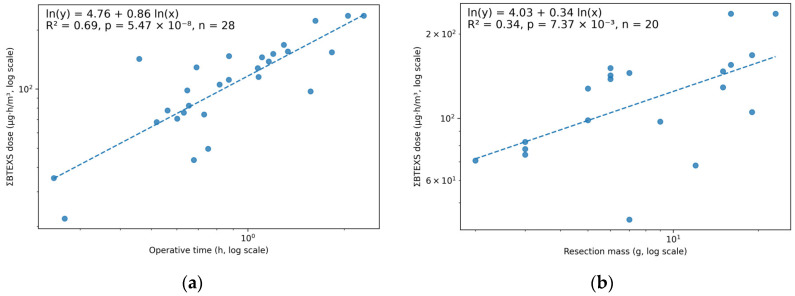
(**a**) Log–log relationship between ΣBTEXS cumulative exposure (dose index, µg·h/m^3^) and operative time during TURBT (*n* = 28). (**b**) Log–log relationship between ΣBTEXS cumulative exposure (dose index, µg·h/m^3^) and resection mass during TURBT (*n* = 20). Points represent individual procedures, and dashed lines indicate the fitted log–log regressions. Abbreviations: ΣBTEXS, sum of benzene, toluene, ethylbenzene, xylenes, and styrene (toluene-equivalents); TURBT, transurethral resection of bladder tumor.

In log–log regression, ΣBTEXS dose scaled with operative time (β = 0.86; 95% CI 0.63–1.10; R^2^ = 0.69; *n* = 28) (Figure 3a). In the mass-available subset (*n* = 20), ΣBTEXS dose also scaled with resection mass (β = 0.34; 95% CI 0.10–0.58; R^2^ = 0.34) (Figure 3b). Operative time was positively correlated with resection mass (Spearman’s ρ = 0.58, *p* = 0.007). In a combined model including both predictors, operative time remained significant (β_time = 0.53, *p* = 0.009), whereas the resection mass term attenuated (β_mass = 0.17, *p* = 0.157), consistent with shared variance between time and tissue mass. Sensitivity analyses addressing missing resection mass showed that restricting to cases with recorded mass yielded a similar time-effect estimate in the time-only model (β_time = 0.67; 95% CI, 0.34–1.01; *n* = 20), supporting the robustness of the dose–time relationship to missing mass.

### 3.5. Hazard Classification and Exposure Benchmarks

Hazard classification (IARC) and selected occupational exposure benchmarks (NIOSH/OSHA) for target analytes relevant to TURBT surgical smoke are summarized in Table 4.

## 4. Discussion

In a prospective, paired design, surgeon breathing-zone concentrations were higher than intraoperative background concentrations for all target analytes, with huge contrasts for aromatic VOCs (Table 2; Figure 2). Breathing-zone TVOC was 4.31-fold higher than background (GMR 4.31; 95% CI 2.92–6.38), and ΣBTEXS was 2.10-fold higher (1.69–2.60); styrene showed the most significant contrast (GMR 8.51; 6.25–11.60), whereas formaldehyde showed only a modest elevation (GMR 1.20; 1.07–1.35). A cumulative dose index increased with operative time (e.g., ΣBTEXS dose Spearman ρ = 0.80, *p* < 0.001) and was also correlated with resection mass, where available (ρ = 0.62, *p* = 0.0038) (Table 3). Together, these findings demonstrate that, even under routine positive-pressure operating room ventilation at approximately 20–25 air changes per hour and in the absence of a dedicated local smoke-evacuation system, proximity to the energy–tissue interface remains the dominant determinant of breathing-zone volatile organic compound exposure during endoscopic bladder tumor resection.

Styrene is a commonly reported component of surgical smoke and has been detected during electrocautery of various tissues, with tissue-dependent VOC profiles that can include styrene alongside other aromatics [22]. At the same time, styrene is also the principal monomer released during thermal degradation of polystyrene and other styrenic plastics; therefore, polymers and disposable materials in the operative field could contribute under sufficient heating [23]. Because we did not perform source-specific sampling, we cannot distinguish tissue-derived styrene from potential device/material contributions; however, the strong breathing-zone enrichment observed here supports a near-field source during active energy use and reinforces the rationale for source-capture controls.

Our results reinforce and extend prior urologic work on composition and proximity. TURP/vaporization studies identified carcinogenic alkenes and nitriles [7]. Comparative analyses have reported more diverse and more hazardous VOCs during TURBT than TURP—notably BTEX [8]. Additional reports have detected toxicants across transurethral resection and vaporization of the prostate (TURVP) and high-performance system (HPS) laser procedures, with some dependence on irrigant [9]. This literature aligns with our observation of elevated BTEX and styrene in the surgeon’s breathing zone. Short-range hazard has long been evident in endoscopic urology: CO levels reached very high peaks (frequently near the monitor ceiling) 15 cm above the resectoscope and decayed quickly with scope withdrawal, underscoring the critical importance of capture geometry and activation timing [6]. Time–distance mapping in a urologist’s OR documented “unhealthy” to worse PM 2.5 spikes at the surgeon’s position within seconds, with wall suction reducing but not eliminating exposure [15]. Our study extends these proximity effects to VOCs and quantifies how time and tissue mass amplify dose.

Although formaldehyde showed a statistically significant breathing-zone elevation in our series (GMR 1.20), the magnitude was small compared with BTEX and styrene (Table 2). This pattern contrasts with some MIS/gynecologic contexts (e.g., loop electrosurgical excision procedure [LEEP]), where formaldehyde within a more enclosed space can become relatively concentrated before filtration [24]. In laparoscopic simulations and clinical reports, built-in filter ports reduce multiple VOCs but may be less effective for formaldehyde, suggesting target-specific sorbent and flow-path refinements [12,24]. Open transurethral irrigation likely promotes dilution and transport, potentially limiting breathing-zone enrichment for this analyte under our OR conditions.

Beyond gases, semi-volatile pyrolysis products from urologic tissues can be biologically active. For example, cholesta-3,5-diene generated from pyrolyzed prostate tissue was identified as a dominant particulate constituent and demonstrated cytotoxicity in primary human oral keratinocytes, supporting biologic plausibility for non-VOC hazards in transurethral smoke [25]. Although our study focused on chemicals, viral nucleic acids—and, in the case of human papillomavirus (HPV), documented transmission via plume—have been reported. A 2021 systematic review concluded that HPV is the pathogen with the clearest occupational signal in plume; importantly, with appropriate capture/filtration, MIS does not appear to increase infectious risk compared with open procedures [26].

From an occupational-health perspective, several analytes relevant to the TURBT plume have International Agency for Research on Cancer (IARC) classifications (Table 4): benzene (Group 1) [27], formaldehyde (Group 1) [28], styrene (Group 2A) [29], and ethylbenzene (Group 2B) [30]. Although our measurements emphasize relative breath-zone elevations rather than threshold exceedances for any single compound, the mixture, peakiness, and repeated operative exposures argue for a precautionary approach that prioritizes engineering controls. This is underscored by stringent benchmarks. For example, the National Institute for Occupational Safety and Health (NIOSH) recommended exposure limit (REL) for formaldehyde is 0.016 ppm as a 10 h time-weighted average (TWA) with a 0.1 ppm 15 min ceiling [31]. In contrast, the Occupational Safety and Health Administration (OSHA) legal permissible exposure limit (PEL) remains 0.75 ppm TWA with a 2 ppm short-term exposure limit (STEL) [32].

To facilitate interpretation against these ppm-based benchmarks, we converted the breathing-zone geometric mean concentrations in Table 2 to approximate ppm at 25 °C and 1 atm (ppm ≈ (mg·m^−3^ × 24.45)/molecular weight). This yields ~0.0015 ppm benzene, 0.016 ppm toluene, 0.0024 ppm ethylbenzene, 0.0071 ppm total xylenes, 0.0016 ppm styrene, and 0.0090 ppm formaldehyde. These task-based averages are below OSHA PELs; however, OELs are defined as shift-based TWAs and/or short-term ceilings, and our integrated sampling does not capture instantaneous peak concentrations or cumulative exposure across multiple procedures in a shift.

Although occupational exposure limits are useful regulatory benchmarks, several key VOCs in surgical smoke (e.g., benzene and formaldehyde) are carcinogenic. They are often considered to have no clearly defined “safe” threshold. To provide a screening-level quantitative context aligned with the ALARA principle, we estimated excess lifetime cancer risk using inhalation unit risk (IUR) values. IUR is defined as the upper-bound increased lifetime cancer risk from continuous inhalation of 1 µg/m^3^ over 70 years [33]. Using the measured breathing-zone geometric mean concentrations in this study (benzene 4.64 µg/m^3^; formaldehyde 11.02 µg/m^3^) and EPA IUR values (benzene 2.2–7.8 × 10^−6^ per µg/m^3^; formaldehyde 1.1 × 10^−5^ per µg/m^3^), the corresponding continuous lifetime risks would be on the order of 10^−5^–10^−4^ [33,34]. Because TURBT exposure is intermittent, the risk for a given individual will scale approximately with the cumulative fraction of lifetime spent under these conditions (risk ≈ IUR × C × hours_exposed/613,200 h). Under this approach, the incremental lifetime cancer risk per 1000 h of exposure at the measured GM concentrations would be ~6 × 10^−8^ for benzene (using the upper IUR) and ~2 × 10^−7^ for formaldehyde. These calculations are screening-level and do not capture peak exposures, co-exposure to other irritants, or inter-individual susceptibility. Still, they underscore that exposure should be minimized even when concentrations are below occupational limits.

Consistent with AORN and NIOSH, LEV at the source (ULPA + activated carbon) should be used routinely, activated before energy delivery, and maintained briefly after cessation to capture early spikes [10,11,12,35,36]. In MIS settings, built-in filter/exsufflation devices are reasonable adjuncts while recognizing potential formaldehyde limitations that warrant device optimization [12,24]. When a residual plume persists, fit-tested N95 (or higher) respirators provide superior submicron filtration compared with surgical masks, which show substantial penetration/leakage for ultrafine particles [37,38]. Personal protective equipment complements, but does not replace, engineering controls. Because cumulative VOC dose scaled with operative time and resection weight in our data, practical steps include efficient resection strategies, staging for large tumor burdens, and meticulous optimization of capture position and flow at the scope–tissue interface (especially during the first seconds of activation, when peaks are typically observed) [15]. Facility policies should align with guideline recommendations and evolving smoke-evacuation policies in multiple jurisdictions and health systems [39,40].

The strengths of this study include paired, simultaneous breathing-zone and background sampling; a standardized sampling window aligned to operative activity; and targeted quantification of aromatic VOCs and aldehydes relevant to surgical smoke. Limitations include the single-center scope; continuous integrated sampling that yielded case-average (TWA) concentrations and likely smoothened short-lived peak exposures during electrosurgical activation; potential influence from the surgeon’s exhaled breath because the inlet was positioned in the breathing zone; absence of simultaneous CO and size-resolved particle counts; limited analyte panel; and lack of detailed ventilation parameters (e.g., measured air-change rate) for the OR. We also used a composite background from three fixed locations and did not conduct a site-by-site spatial analysis. Future work should integrate real-time monitoring to capture peaks, characterize a broader VOC profile, and evaluate the effectiveness of practical engineering controls (e.g., source capture and filtration) in endoscopic urologic surgery.

## 5. Conclusions

During TURBT, surgeons experience substantially higher VOC concentrations in the breathing zone than background levels—particularly BTEX compounds and styrene—and cumulative doses increase with operative time. This is also associated with resection mass, where available. These findings, together with prior evidence on chemical and particulate hazards in transurethral surgery, reinforce the need for routine, source-level smoke evacuation and filtration in endoscopic urology and a systems approach that prioritizes engineering controls, consistent with modern guidelines.

## Figures and Tables

**Table 1:** Characteristics of TURBT cases and sampling sessions.

Characteristic	Value
TURBT procedures (*n*)	28
Sampling days	10
Operative time (min)	51 [IQR 39–73] (median [IQR]); 59.2 ± 31.0 (mean ± SD); range 15–138
Resection mass (g)	7.0 [IQR 5.0–15.2] (median [IQR]); 9.85 ± 6.43 (mean ± SD); range 2.0–23.0

Abbreviations: TURBT, transurethral resection of bladder tumor; IQR, interquartile range; SD, standard deviation.

**Table 2:** Surgeon breathing-zone concentrations versus intraoperative background concentrations of target analytes during TURBT (paired geometric mean ratios with 95% confidence intervals). Background concentrations represent the per-procedure geometric mean across the three background sites (sites 1, 3, and 4). TVOC refers to ISO-TVOC (C6–C16, toluene equivalents) where available.

Analyte	Surgeon GM (GSD), µg/m^3^	Background GM (GSD), µg/m^3^	GMR (95% CI)	*p* (Paired *t*-Test on ln Ratios)
TVOC	1201.11 (2.03)	278.51 (2.09)	4.31 (2.92–6.38)	<0.0001
ΣBTEXS (Benzene + Toluene + Ethylbenzene + Xylenes + Styrene)	118.85 (1.39)	56.64 (1.69)	2.10 (1.69–2.60)	<0.0001
Benzene	4.64 (1.52)	2.92 (1.50)	1.59 (1.39–1.82)	<0.0001
Toluene	60.82 (1.54)	32.53 (1.88)	1.87 (1.46–2.40)	<0.0001
Ethylbenzene	10.38 (1.38)	6.52 (1.59)	1.59 (1.32–1.92)	<0.0001
Xylenes (Total)	31.01 (1.64)	12.65 (1.54)	2.45 (1.91–3.15)	<0.0001
Styrene	6.77 (1.77)	0.80 (1.95)	8.51 (6.25–11.60)	<0.0001
Formaldehyde	11.02 (1.32)	9.16 (1.59)	1.20 (1.07–1.35)	0.0023

Abbreviations: TURBT, transurethral resection of bladder tumor; GM, geometric mean; GSD, geometric standard deviation; GMR, geometric mean ratio; CI, confidence interval; TVOC, total volatile organic compounds; ΣBTEXS, sum of benzene, toluene, ethylbenzene, xylenes, and styrene (toluene-equivalents).

**Table 3:** Associations between cumulative exposure (dose index, µg·h/m^3^) and operative time or resection mass (Spearman correlation).

Dose Metric (µg·h/m^3^)	*n* (Time)	Spearman ρ vs. Operative Time	*p* (Time)	*n* (Mass)	Spearman ρ vs. Resection Mass	*p* (Mass)
ΣBTEXS dose	28	0.80	<0.001	20	0.62	0.0038
TVOC dose	28	0.52	0.0047	20	0.39	0.09
Benzene dose	28	0.50	0.0066	20	0.35	0.134
Toluene dose	28	0.61	<0.001	20	0.42	0.067
Ethylbenzene dose	28	0.81	<0.001	20	0.59	0.0063
Xylenes dose	28	0.71	<0.001	20	0.56	0.0098
Styrene dose	28	0.79	<0.001	20	0.59	0.0061
Formaldehyde dose	28	0.79	<0.001	20	0.48	0.032

Abbreviations: ΣBTEXS, sum of benzene, toluene, ethylbenzene, xylenes, and styrene (toluene-equivalents); TVOC, total volatile organic compounds; ρ, Spearman’s rank correlation coefficient.

**Table 4:** Hazard classification (IARC) and selected occupational exposure benchmarks (NIOSH/OSHA) for target analytes relevant to TURBT surgical smoke.

Substance	IARC Group	NIOSH REL	OSHA PEL	Key Hazards (Inhalation)
Benzene	Group 1 (carcinogenic to humans)	0.1 ppm TWA; 1 ppm STEL	1 ppm TWA; 5 ppm STEL	Hematotoxicity (bone marrow); leukemia risk; CNS symptoms
Formaldehyde	Group 1 (carcinogenic to humans)	Carcinogen: 0.016 ppm TWA; 0.1 ppm (15 min ceiling)	0.75 ppm TWA; 2 ppm STEL	Strong mucosal/respiratory irritant; asthma-like symptoms; nasal cancer risk
Styrene	Group 2A (probably carcinogenic to humans)	50 ppm TWA; 100 ppm STEL	100 ppm TWA (also ceiling/peak in Table Z-2)	Eye/respiratory irritation; CNS depression; possible liver/reproductive effects
Ethylbenzene	Group 2B	100 ppm TWA; 125 ppm STEL	100 ppm TWA	Eye/respiratory irritation; CNS symptoms
Toluene	Group 3 (not classifiable as to carcinogenicity to humans)	100 ppm TWA; 150 ppm STEL	200 ppm TWA (also ceiling/peak in Table Z-2)	CNS effects (headache, dizziness); irritation; liver/kidney effects
Xylenes (o/m/p)	Group 3 (not classifiable as to carcinogenicity to humans)	100 ppm TWA; 150 ppm STEL	100 ppm TWA	Eye/respiratory irritation; CNS depression

Abbreviations: IARC, International Agency for Research on Cancer; NIOSH, National Institute for Occupational Safety and Health; OSHA, Occupational Safety and Health Administration; REL, recommended exposure limit; PEL, permissible exposure limit; TWA, time-weighted average; STEL, short-term exposure limit; CNS, central nervous system.

## Data Availability

The data presented in this study are not publicly available due to privacy and ethical restrictions related to intraoperative occupational exposure measurements but are available from the corresponding author upon reasonable request.

## Abbreviations

The following abbreviations are used in this manuscript:

BTEX	benzene, toluene, ethylbenzene, and xylenes
BTEXS	benzene, toluene, ethylbenzene, xylenes, and styrene
DNPH	2,4-dinitrophenylhydrazine
GC–MS	gas chromatography–mass spectrometry
LEV	local exhaust ventilation
LOQ	limit of quantitation
TURBT	transurethral resection of bladder tumor
TVOC	total volatile organic compounds
ULPA	ultra-low particulate air
VOC	volatile organic compounds
ΣBTEXS	sum of benzene, toluene, ethylbenzene, xylenes, and styrene (toluene-equivalents)
